# Three-Dimensional Visualization System for Vitreoretinal Surgery: Results from a Monocentric Experience and Comparison with Conventional Surgery

**DOI:** 10.3390/life13061289

**Published:** 2023-05-31

**Authors:** Fabrizio Giansanti, Cristina Nicolosi, Daniela Bacherini, Federica Soloperto, Federica Sarati, Dario Giattini, Giulio Vicini

**Affiliations:** 1 Eye Clinic, Neuromuscular and Sense Organs Department, Careggi University Hospital, 50134 Florence, Italy; 2 Department of Neurosciences, Psychology, Drug Research and Child Health, University of Florence, 50121 Florence, Italy; 3 Azienda USL Toscana Nord Ovest, 56121 Pisa, Italy

**Keywords:** 3D surgical viewing system, vitreoretinal surgery, vitreoretinal diseases, retinal detachment, macular hole, epiretinal membrane

## Abstract

Purpose: To describe the experience of our centre (Careggi University Hospital, Florence, Italy) in using a heads-up three-dimensional (3D) surgical viewing system in vitreoretinal surgery, making a comparison with the conventional microscope surgery. Methods: We retrospectively analyzed data taken from 240 patients (240 eyes) with surgical macular diseases (macular hole and epiretinal membrane), retinal detachment or vitreous hemorrhage who underwent vitreoretinal surgeries, by means of the NGENUITY 3D Visualization System (Alcon Laboratories Inc., Fort Worth, TX, USA), in comparison with 210 patients (210 eyes) who underwent vitreoretinal surgeries performed using a conventional microscope. All surgeries were performed with standardized procedures by the same surgeons. We analyzed data over a follow-up period of 6 months, comparing the surgical outcomes (best-corrected visual acuity, anatomical success rate and postoperative complication rate) between the two groups. Results: the 3D group included 74 patients with retinal detachment, 78 with epiretinal membrane, 64 with macular hole and 24 with vitreous hemorrhage. There were no significant differences in the demographic and clinical characteristics between the 3D group and the conventional group. We found no significant differences in outcome measures at three and six months follow-up between the two groups (*p*-value ≥ 0.05 for all comparisons). Surgery durations were similar between the two groups. Conclusions: In our experience, a heads-up 3D surgical viewing system provided comparable functional and anatomical outcomes in comparison with conventional microscope surgery, proving to be a valuable tool for vitreoretinal surgery in the treatment of different retinal diseases.

## 1. Introduction

Since the introduction of three-dimensional (3D) visualization systems in vitreoretinal surgery, it has been increasingly employed by vitreoretinal surgeons, bringing a new ophthalmic microsurgery experience with periodically updated high-definition screen devices [1,2,3].

A 3D surgery system utilizes two high-definition dynamic cameras to record the image from different microscope viewing angles and a high-definition 3D display to receive a processed image [4]. It allows the surgeon to operate in a more comfortable position than a traditional microscope, meaning that they can raise their head up (the 3D system is, consequently, also known as a heads-up visualization system) and directly look at the surgical field on a large, high-definition 3D screen rather than a microscope eyepiece [1,4,5].

Some potential advantages of a 3D heads-up visualization system in comparison with a traditional microscope have been reported in different studies and include a higher-resolution visualization of the surgical field, higher magnification and digital processing of the image, more comfortable ergonomics for surgeons, and better surgical training, as all the people in the operating room can observe the same surgical field on the 3D display. The 3D viewing system enables the visualization on a single digital display of the imaging components (as intraoperative optical coherence tomography) and live details, such as the vitrectomy parameters, overlayed on the surgical field. Moreover, it may allow for the use of lower endoillumination parameters compared to a conventional microscope, compensated by a digital amplification of the camera signals, and possibly causing less retinal phototoxicity [1,5,6,7,8,9,10,11,12,13,14,15,16,17,18].

Different studies have been published on the application of 3D visualization systems in vitreoretinal surgery [1,5,17,19,20,21,22,23,24,25,26,27,28,29,30], but only a few studies have included large numbers of patients [24,25,26,27,29,30].

The aim of our study is to describe the experience of our center (Careggi University Hospital, Florence, Italy) concerning the application of a 3D visualization system in vitreoretinal surgery in a large series of patients affected by vitreoretinal surgical diseases when compared to conventional microscope surgery.

## 2. Materials and Methods

### 2.1. Subjects

We conducted a retrospective, comparative study at a single center (Ophthalmology department of Careggi University Hospital, Florence, Italy) over a period of 3 years. We analyzed patients affected by vitreoretinal diseases operated between January 2019 and January 2022, using either the NGENUITY System (3D group) or a traditional microscope (TM group). The surgical indicators included epiretinal membranes (ERMs), full-thickness macular holes (MHs), vitreous hemorrhage (VH), and rhegmatogenous retinal detachment (RRD).

A total of 450 patients were included in the study, distributed into the group undergoing surgery with the NGENUITY System (n = 240) and the group undergoing surgery that used conventional microscopy (n = 210). 

The 3D group included 74 (30.8%) patients with RRD, 78 (32.5%) with idiopathic ERM, 64 (26.7%) with MH, and 24 (10%) with VH. The TM group included 65 (30.9%) patients with RRD, 67 (31.9%) with idiopathic ERM, 52 (24.8%) with MH, and 26 (12.4%) with VH. One hundred forty-four (60%) patients in the 3D group and 111 (52.9%) patients in the TM group underwent combined surgery with phacoemulsification and capsular bag intraocular lens implantation.

### 2.2. Methodology

Patients operated upon with the use of the 3D visualization system between January 2019 and January 2022, with a minimum 6 months follow-up, which were included in the study and were compared to patients operated upon using a traditional microscope, with a minimum 6 months follow-up, who were matched for pathology, sex, and age. The medical records of patients who underwent pars plana vitrectomy (PPV) for vitreoretinal diseases with the use of a 3D visualization system (3D group) or a traditional microscope (TM group) were reviewed. Patients with a postoperative follow-up of 6 months at least were included in the study. The review of medical records was approved by the Local Ethics Committee and adhered to the tenets of the declaration of Helsinki. All of the patients signed a written informed consent, agreeing to participate.

**Figure 1:**
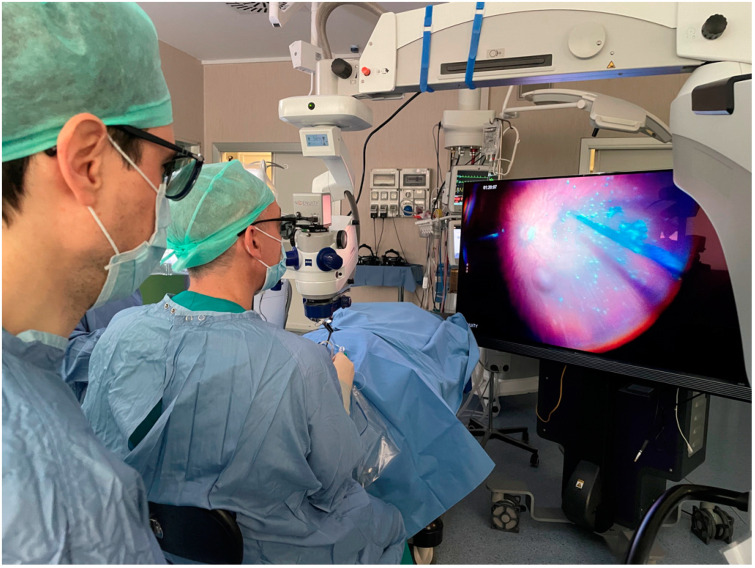
Our operating theatre configuration with the NGENUITY 3D Visualization System.

The surgeries were performed under local retrobulbar or general anesthesia by experienced vitreoretinal surgeons with standardized procedures. The surgical techniques did not differ between the 3D and TM groups. Three-port 25- or 23-gauge pars plana vitrectomy was performed with a CONSTELLATION Vision System (Alcon Laboratories, Fort Worth, TX, USA) using an aperture diaphragm of almost 1/2 to limit endoillumination exposure and optimize the visualization. All the surgical procedures were performed using an OPMI LUMERA 700 surgical microscope (Carl Zeiss Meditec, Jena, Germany) and a non-contact wide-angle RESIGHT viewing system (Zeiss, Oberkochen, Germany). The microscope eyepieces remained mounted for the TM group, and they were disassembled and replaced with the NGENUITY 3D visualization system (Alcon Laboratories), with the NGENUITY v1.4.31 software version, mounted on the microscope for the 3D group (Figure 1). The surgeon, the assistants, and the theatre nurses wore passive circularly polarized glasses to look at the surgical field on the 3D display.

Endoillumination light levels were initially set to 30–40% of maximum output for patients in the 3D group and 70–80% in the TM group, respectively. During the surgery, these levels were adjusted to optimize retinal visualization if necessary. Intraoperative Optical Coherence Tomography (OCT) was used during vitreomacular interface surgeries. Color filters were adjusted according to phacoemulsification and vitreoretinal surgery. The pre- and postoperative schemes were the same in both groups, and all employed a 23- or 25-gauge three-port pars plana vitrectomy technique. The surgical procedures varied based on the diagnosis. The inverted internal limiting membrane (ILM) flap technique was applied to eyes with MHs at the surgeon’s discretion. Endolaser was employed in the occurrence of retinal tears, RRD, and proliferative diabetic retinopathy. Fluid–air exchanges were performed when indicated. Endotamponade was performed with a balanced salt solution, air, gas, or silicon oil, which were employed according to the diagnosis. All the patients were administered topical antibiotics, corticosteroids, and anti-inflammatory eyedrops for 2 to 4 weeks postoperatively. The computerized operating registers extracted from our operating room report program allowed us to recover precise information concerning the surgical procedures and their duration, recorded by the equipe participating in each surgery. Two investigators (F.So. and F.Sa.) extracted the baseline and outcome data. The following patient information was extrapolated from the operating registers and from the medical records: age, gender, baseline lens status, diagnosis, surgical indication, ocular history, baseline best-corrected visual acuity (BCVA), 6-month postoperative BCVA, surgery duration, baseline anatomical data (macular hole diameter, ERM baseline central macular thickness, and RRD macular involvement) and surgical outcome data at 6 months from surgery. Pre- and postoperative BCVA was expressed as a decimal. Surgery duration was measured in minutes and was defined as the operation time from the first incision to the final removal of the blepharostat. MH diameter was defined by structural OCT, drawing with the caliper function a horizontal line connecting the two closest foveal points. ERM baseline central macular thickness was defined as the mean thickness within the central 1000 μm diameter area, calculated with the use of the OCT software on a thickness map. Macula-on RRD was defined as a condition where the fovea was not involved at the time of presentation.

### 2.3. Analysis

The surgical anatomical outcomes analyzed differed according to the surgical indication. They included the rate of MH closure (%), the rate of ERM removal (%), the rate of RRD reattachment (%), and the rate of VH clearing (%).

MH closure was defined as the flattening of MH with the absence of a neurosensory defect at the fovea. ERM removal was defined as complete ERM removal without signs of recurrence. RRD reattachment was defined as the complete reattachment of the retina. VH clearing was defined as the complete removal of blood within the vitreous cavity.

The structural and functional outcome endpoints used for effectiveness comparisons were based on anatomical outcomes, changes in BCVA, and surgery duration.

Statistical analysis was performed using IBM SPSS Statistics (IBM Corporation, Armonk, NY, USA) software for Mac (Version 26.0). Demographic and clinical data of the two groups, as well as the surgical outcomes, were compared using a two-tailed Student’s *t*-test or Chi-square test with 95% confidence intervals. Normal distribution of the data was determined using the Shapiro–Wilk test. The statistical significance was defined as a *p*-value of <0.05.

## 3. Results

No significant differences were found in the demographic and clinical data (age, gender, baseline lens status, surgical indications) between the 3D and TM groups. Additionally, the baseline anatomical characteristics of the different retinal diseases studied (MH diameter, ERM baseline central macular thickness, and RRD macular involvement) did not significantly differ between the patients in the 3D and TM groups. The demographic and clinical characteristics of the patients included in the study are summarized in Table 1. 

No major intraoperative complications were encountered in both groups. No statistically significant differences were identified in outcomes analyzed during surgery follow-up between the 3D and TM groups. The surgical results data in 3D and TM groups are summarized in Table 2.

The rate of retinal reattachment of RRD in our study series was 92.1%, with a reattachment rate of 93.8% in the 3D group and 90.8% in the TM group (*p*-value = 0.59). The rate of MH closure at 3 months was 94%: 93.8% in the 3D group and 94.2% in the TM group (*p*-value = 0.91). The ERM removal was successful in both groups. Successful ERM removal was obtained in 100% of patients in both groups. Baseline BCVA was 0.36 in the 3D group and 0.41 in the TM group. There were no significant differences in the baseline and postoperative BCVA values between the two groups (*p*-value = 0.67 and 0.12, respectively). Both groups showed significant improvements in the mean BCVA at 6 months from surgery (*p*-value < 0.001). 

Surgery durations were similar between both groups: 60.7 min in the 3D group and 61 min in the TM group (*p*-value = 0.46). Analysis of the different disease subgroups showed no significant differences (*p*-value ≥ 0.05 for all comparisons).

## 4. Discussion

In our study, we compared the outcome of 240 surgeries performed with the 3D Visualization System to 210 surgeries performed using conventional microscopy, selecting the same pathologies. We did not find any significant differences in overall visual outcomes (pre- and postoperative BCVA), anatomical outcomes (such as the removal of ERM, MH closure, retinal reattachment in RRD, and VH clearing), and surgery durations between the two groups. These results are in agreement with other studies, showing no significant differences in safety, anatomical outcomes and visual prognosis when comparing the usage of 3D visualization systems and conventional microscopes in the same vitreoretinal surgery techniques [1,20,21,22,23,24,25,26,27,28,29,30].

Regarding MH closure, the closure rate in our series was 93.99%, according to closure rates reported in the literature (90–100%), without significant differences between the two groups [31]. Guber et al. demonstrated a 91.9 µm reduction in central macular thickness at 3 months after vitrectomy in patients affected by primary ERM. The decrease of central macular thickness in ERMs was not statistically different in the two groups and was in line with the decrease in thickness reported at 3 months after vitrectomy in the literature [32].

Three-dimensional surgical visualization systems allow the ophthalmic surgeon to switch traditional microscope eyepieces with cameras transmitting an image on a high-definition display in front of them. Different advantages of a heads-up 3D visualization system over traditional microscopy have been described yet. First of all, the field depth has been reported to be similar or better in 3D systems in comparison to a traditional microscope because of the better light sensitivity of the software and the two high dynamic range cameras; the diaphragm aperture can be reduced, and the field depth increased [20,33,34]. The field depth is greater than the standard analog surgical microscope by 2–3 times if the opening of the NGENUITY system camera is reduced to 30%. This difference is not significant when the zoom level is high [34]. The dynamic range of the surgical images can be expanded by gain, gamma, and tone curve correction, uniformly adjusting the brightness and darkness of the image. High dynamic range cameras may combine multiple images from different points of view to improve the dynamic range balance of bright and dark areas of the same image, but they cannot manage image clouding or general hazes [35,36]. The sharpness is greater than conventional microscopes, and the surgeon requires less effort in terms of accommodation, especially older surgeons who have a smaller accommodative reserve [30]. It has been demonstrated that image-sharpening algorithms may ameliorate the clarity of all objects in the surgical field during combined cataract and vitreoretinal surgery using a 3D visualization system [37]. Image sharpening and color adjustments in real-time can enhance the intraoperative visibility in 3D surgery with the employment of the NGENUITY 3D Visualization System, by improving the contrast and ameliorating the image resolution, by narrowing the point spread function [37]. 

Moreover, the 3D image is achieved through the combination of two high dynamic range camera images, which are processed by algorithms, allowing for the magnification of lower light levels [30]. Endoillumination levels are also reduced, preserving adequate visualization. Therefore, decreased endoillumination reduces retinal light exposure during surgeries and retinal phototoxicity, especially during macular surgery, for example, by keeping the light source at a greater distance from the retina [37]. 

**Figure 2:**
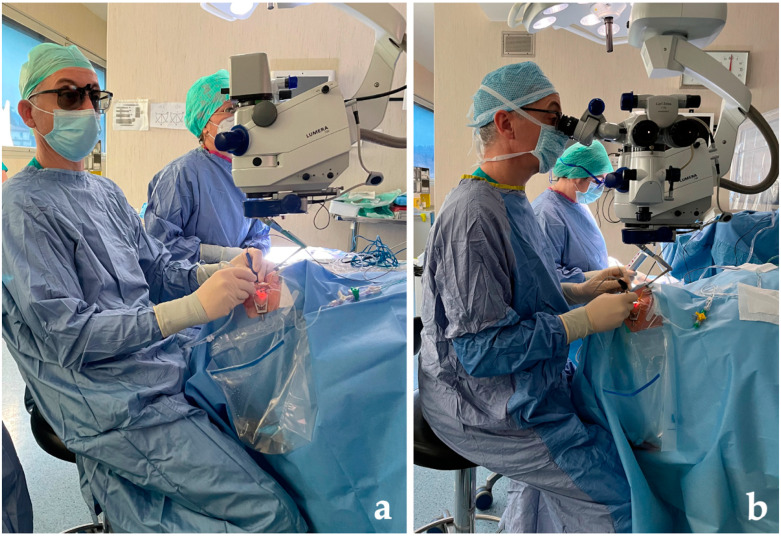
The images show the better ergonomics of the 3D visualization system for the surgeon (**a**), in comparison with the conventional microscopy configuration (**b**).

In relation to the facility of employment of the NGENUITY 3D Visualization System, the opinions of surgeons have been previously analyzed with satisfaction questionnaires by comparing fine surgical tasks [22,38]. These satisfactory questionnaires also showed an improvement in comfort, a more ergonomic position and a reduction in back and neck pain, which is frequently detected among ophthalmologic surgeons [22,38,39]. The different ergonomics and head and neck positions of the ophthalmic surgeon in the employment of both a 3D visualization system and a conventional microscopy configuration are shown in Figure 2.

**Figure 3:**
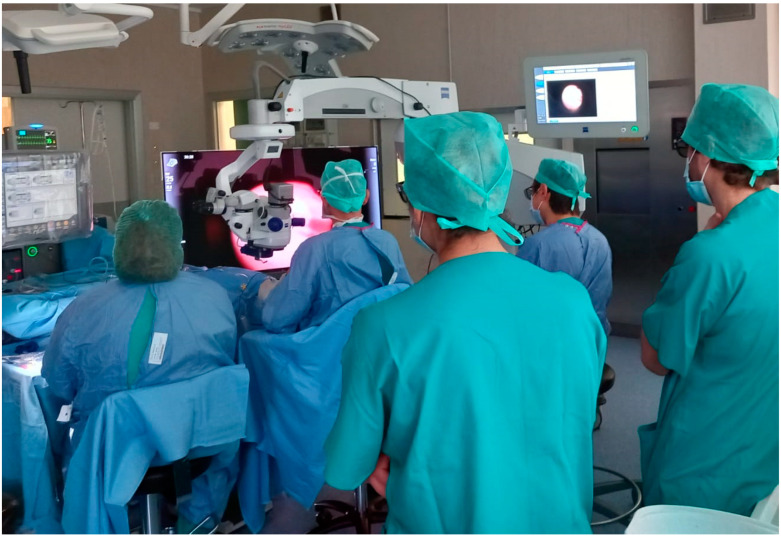
Interns surgical training, as all the people in the operating room can observe the same surgical field on the 3D display.

Regarding surgical training and education, there are some advantages to using a 3D visualization system. All the people present in an operating theatre can look at the same live surgical field image, conversely to the conventional microscope, in which only the first and second operators can look at the surgical field in a high-definition way. Additionally, the first operator can teach more than one trainee intern at the same time, as shown in Figure 3. The 3D image can also be recorded and retransmitted at a distance or live, and surgical video streaming can be achieved in real-time with minimal latency through video capture equipment and video conferencing/streaming software [1,40,41,42].

The employment of heads-up 3D visualization system technology in vitreoretinal surgery has been reported to be effective, but only a few published studies have included large numbers of patients [24,25,26,27,29,30]. Different studies that have compared heads-up 3D viewing system technology with conventional microscopes in vitreoretinal surgery found similar anatomical and functional outcomes in addition to comparable surgical efficiency (Table 3).

Asani et al. compared PPV for rhegmatogenous retinal detachment using either the NGENUITY 3D Visualization System (n = 70) or a standard operating microscope (n = 70), yielding similar results in terms of anatomical and functional outcomes (primary retinal reattachment rate, rate of proliferative vitreoretinopathy, and final BCVA); however, surgery time was slightly longer in the 3D group (REF). Interestingly, this result was evident when looking at the first 35 cases, but it was not reproducible when only comparing the latest 35 cases against each other, suggesting the effect of the learning curve required for the 3D platform [30]. Another study conducted by Talcott et al. indicates a learning curve for the 3D platform. This was a prospective randomized study that reported a series of 39 patients undergoing PPV with peeling for macular pathology, including ERM and MH. Although the overall surgical times were similar, the macular peel times in the 3D group were longer and associated with less ease of use in this study, which may partly be due to a learning curve required for the use of 3D technology [21].

The study conducted by Zhao et al. showed different results, with the duration of ERM or ILM peeling for eyes with ERM and idiopathic MH significantly shorter in the 3D group than in the conventional microscope group. This result was associated with significantly shorter general surgical duration for eyes with ERM and idiopathic MH. The authors suggest that one possible reason could be that the 3D heads-up surgery has the advantage of high image magnification at a wider visual field compared with the conventional microscope, which enables surgeons to view the fine structures of the retina and then perform membrane peeling more precisely [27].

In our series, we found similar values in the duration of surgery, both considering the overall series and the different pathologies, in agreement with most of the literature. Although we did not record the ERM or ILM peeling time in our study, no differences were documented in the mean duration of the complete operations for ERM and MH in the two groups.

In summary, we reported the clinical surgical outcomes of 3D visualization system for vitreoretinal diseases in a large series of patients operated at a single center. In our series, the heads-up 3D visualization system appears to be comparable to traditional surgical microscopy in terms of effectiveness and safety in the treatment of RRD, ERM, MH, and VH. The visual and anatomical outcomes and the surgery duration were not statistically different from those of traditional microscope vitrectomies for the different surgical indicators. Our findings need to be confirmed in further prospective, randomized studies. 

## 5. Conclusions

In conclusion, in our experience, the 3D heads-up visualization system can be considered a valuable and safe tool for vitreoretinal surgery, but further prospective, randomized studies are required to confirm these preliminary findings.

## Figures and Tables

**Table 1:** Demographic and clinical characteristics of the patients included in the study.

	3D Group	Traditional Microscope Group	*p*-Value
Number of patients, n	240	210	
Age, years (mean ± SD)	59.3 ± 12.1	61.7 ±13.3	0.075 *
Sex, n (%)			0.63 **
Male	149 (62.1%)	135 (64.3%)	
Female	91 (37.9%)	75 (35.7%)	
Lens status, n (%)			0.41 **
Phakia	144 (60%)	111 (52.9%)	
Pseudophakia	96 (40%)	99 (47.1%)	
Indications, n (%)			0.86 **
Retinal detachment	74 (30.8%)	65 (30.9%)	
Idiopathic epiretinal membrane	78 (32.5%)	67 (31.9%)	
Macular hole	64 (26.7%)	52 (24.8%)	
Vitreous hemorrhage	24 (10%)	26 (12.4%)	
Macular hole diameter, µm (mean ± SD)	374.2 ± 125.3	392.75 ± 139.3	0.45 *
Epiretinal baseline central macular thickness, µm (mean ± SD)	438.75 ± 125.8	441.67 ± 81.2	0.52 *
Retinal detachment macular involvement, n (%)	39 (52.7%)	32 (49.2%)	0.68 **
Baseline decimal BCVA (mean)	0.36	0.41	0.67 *

BCVA = best-corrected visual acuity; * Student’s *t*-test; ** Chi-square test.

**Table 2:** Surgical results of patients included in the study.

	3D Group (N = 240)	Traditional Microscope Group (N = 210)	*p*-Value
Post-op decimal BCVA (mean)	0.53	0.57	0.12 *
Surgery time, minutes (mean)	60.7	61.0	0.46 *
Retinal detachment time, minutes (mean)	69	67	0.26 *
Idiopathic epiretinal membrane time, minutes (mean)	57.6	56.6	0.74 *
Macular hole time, minutes (mean)	56.14	58.1	0.86 *
Vitreous hemorrhage time, minutes (mean)	57.05	63.0	0.16 *
Surgical outcome ***			
ERM removal, %	100	100	-
MH closure, %	93.8	94.2	0.91 **
Retinal reattachment, %	93.2	90.8	0.59 **
VH clearing, %	95.8	96.1	0.95 **

BCVA = best-corrected visual acuity; * Student’s *t*-test; ** Chi-square test; *** at 6-month follow-up.

**Table 3:** Studies reported in literature regarding the comparison of heads-up 3D visualization system technology with conventional microscope in vitreoretinal surgery.

Authors	Number of Patients 3D Group/CM Group	Type of Treatment/ Surgical Indication	Outcome	Results
Kumar et al. [23]	25/25	PPV with multilayered inverted ILM membrane flap technique and 20% SF6 for FTMH	Pre- and postoperative BCVA, macular hole index, total surgical time, total ILM peel time, number of flap initiations, duration of Brilliant Blue G dye exposure, and illumination intensity	Comparable clinical outcomes. Illumination intensity of microscope and endoillumination were significantly less in the 3D group
Talcott et al. [21]	23/16	PPV for ERM and FTMH	Total operative time, macular peel time, surgeon rating of viewing system ease of use, minimum required endoillumination, intraoperative complication rate, and postoperative BCVA	No significant difference in overall operative time, but macular peel time was significantly longer using 3D HUD and associated with less ease of use. The minimum required endoillumination was significantly lower with 3D HUD. No significant differences in BCVA and complication occurrence
Zhang et al. [24]	124/202	PPV for RRD, FTMH, ERM, VH, VO, SOR, and MF	Pre- and postoperative BCVA, ERM removal VH clearing, MH closure, RD reattachment, MF resolution, SOR success, VO clearing, operation time, postoperative complications occurrence	Comparable visual and anatomical outcomes without a significant difference in the rate of complications
Palácios et al. [33]	94/94	PPV for RRD and MH	Surgeon preference was assessed using a questionnaire, anatomical success rate	Comparable anatomical outcomes
Asani et al. [30]	70/70	PPV for RRD	Primary retinal reattachment rate, rate of proliferative vitreoretinopathy, final BCVA, duration of surgery	Comparable anatomical and functional outcomes. Duration of surgery was significantly longer in the 3D group, an effect which, however, vanished after a “learning curve” of the first 35 eyes
Kantor et al. [26]	131/96	PPV for RRD, FTMH, and ERM	Primary endpoints: recurrence rates of RD, FTMH closure rates, reduction in central macular thickness in ERMs at 3 months after surgery. Secondary endpoints: surgery durations, 3-month postoperative BCVA	Comparable visual and anatomical outcomes
Zhao et al. [27]	220/242	PPV for RRD, TRD, FTMH, ERM, VMT, VH, VO, SOR, and MF	BCVA, primary anatomical success (varied according to the surgical indicators), general surgical duration, duration of specific surgical steps, perioperative complications, and satisfaction feedback from the surgical team	Comparable efficacy and safety. Shorter duration of ERM or ILM peeling for the 3D HUD group with significantly shorter general surgical duration for ERM and MH surgery. Better surgical team satisfaction
Nowomiejska et al. [28]	26/56	PPV combined with cataract surgery for RRD	BCVA, surgery duration, rate of postoperative complications	No significant differences in surgery duration, rate of complications, and functional results
Zeng et al. [29]	50/138	PPV alone or combined PPV and scleral buckle for RRD	Anatomic success rate, rate of postoperative proliferative vitreoretinopathy, surgery duration	Anatomical and functional outcomes and surgical efficiency comparable in the two groups

PPV: pars plana vitrectomy; ILM: internal limiting membrane; FTMH: full-thickness macular hole; BCVA: best-corrected visual acuity; ERM: epiretinal membrane; 3D HUD: 3D heads-up display; RRD: rhegmatogenous retinal detachment; VH: vitreous hemorrhage; VO: vitreous opacities; SOR: silicone oil removal; MF: pathologic myopic foveoschisis; TRD: tractional retinal detachment; VMT: vitreomacular traction syndrome.

## Data Availability

The data presented in this study are available upon request from the corresponding author.
